# Urine l-carnitine excretion in hypertensive adolescents

**DOI:** 10.1007/s11845-014-1091-6

**Published:** 2014-03-01

**Authors:** A. Kępka, E. Kuroczycka-Saniutycz, S. Chojnowska, R. Fiłonowicz, A. Korzeniecka-Kozerska, A. Wasilewska

**Affiliations:** 1 Department of Biochemistry, Radioimmunology and Experimental Medicine, The Children’s Memorial Health Institute, Warsaw, Poland; 2 Department of Pediatrics and Nephrology, Medical University of Białystok, 17 Waszyngtona street, 15-274 Białystok, Poland; 3 Medical Institute, College of Computer Science and Business Administration, Łomża, Poland

**Keywords:** Urinary l-carnitine, Hypertension, Hyperuricemia, Adolescents

## Abstract

**Aim:**

This study was performed to test the hypothesis that urinary levels of l-carnitine and its derivatives are enhanced in children and adolescents with hypertension and also check if analyzed parameters may serve as early markers of subclinical renal damage.

**Methods:**

The study included 112 children and adolescents (67 males and 45 females) aged median 10–18 years. Participants were divided into two groups: HT—64 subjects with confirmed primary hypertension and R—reference group—48 subjects with white-coat hypertension.

Urinary Free and Total l-carnitine were determined by the enzymatic method of Cederblad. The l-carnitine levels were expressed as urinary ratio in micromole per gram creatinine (μmol/g).

**Results:**

The urinary excretion of Total and Free l-carnitine was significantly higher in hypertensive adolescents in comparison to reference group—white coat hypertension. Other important findings were positive correlations between Free l-carnitine/cr., Total l-carnitine/cr. ratio and serum uric acid level, serum cholesterol level and systolic blood pressure.

**Conclusion:**

The results of this study do not explain the increased urine levels of l-carnitine. The most likely reason for excessive urinary loss was disturbed renal tubular reabsorption. It is possible to hypothesize that in hypertensive adolescents subclinical kidney dysfunction occurs. It is proposed that studies examining the concurrent plasma and urine concentration of l-carnitine and correlation with acknowledged proximal tubular markers are needed.

## Introduction

The prevalence of hypertension (HT) is increasing among children and adolescents. It has become a significant public health issue, because it has been associated with significant risk for future cardiovascular disease and target organ damage. Many factors have been associated with the development of HT so far. One of the important metabolic abnormalities in children and adolescents with primary HT appears to be elevated serum uric acid (SUA) levels. Many large epidemiologic studies have demonstrated that an elevated SUA level is associated with HT [1–5]. Hypertension plays a pivotal role in the progression of renal failure.

In recent years, the role of l-carnitine (LC) and its derivatives in the pathogenesis of hypertension has been the subject of considerable attention. Experimental and clinical data support that LC treatment exerts beneficial effects on several disorders, including hypertension [6] and chronic kidney diseases [7]. Studies in humans have shown that LC supplementation may be effective in reducing arterial blood pressure in hypertensive patients [8]. It is thought that low level of serum LC in patients with hypertension may be associated with a higher urinary excretion of LC, which may be caused by renal injury in the course of HT.

l-carnitine is essential for the transport of long-chain fatty acids into the mitochondrial matrix, where they can be metabolized by β-oxidation [9]. LC is derived from both dietary sources (75 %) and endogenous biosynthesis (25 %) in human body [10]. LC can be acylated to form acylcarnitines but is otherwise not metabolized [9, 11].

Its elimination occurs as carnitine or acylcarnitine primarily via urinary excretion [9, 11]. The kidney is an important organ in LC metabolism (after glomerular filtration, it reabsorbs 98–99 % of LC in the proximal tubule and it is involved in LC synthesis). Foster et al. [12] demonstrated that urine excretion of LC was increased by 150 % in hypertensive rats. In the available literature, there are no data regarding the excretion of LC and its derivatives in patients with hypertension.

This study was performed to test the hypothesis that urinary levels of LC and its derivatives are enhanced in children and adolescents with hypertension and also check if analyzed parameters may serve as early markers of subclinical renal damage.

## Patients and methods

This was a prospective cohort study of hypertensive children and adolescents. The study included 112 subjects (45 girls and 67 boys) aged 10–18 years, who were appointed to our unit (Department of Pediatrics and Nephrology, The Medical University of Białystok, Poland) between January 2011 and January 2013 to confirm or rule out hypertension. On the basis of ABPM, the examined adolescents were divided into two groups: HT—64 subjects with confirmed primary hypertension and R—reference group—48 subjects with white-coat hypertension. The subjects from reference group were referred to our Department by general practitioners after finding the elevated (the 95th percentile or higher) causal BP in primary care office, but the values of blood pressure were completely normal in ABPM (mean systolic and diastolic daytime and nighttime blood pressure levels less than the 90th percentile for age, sex and height and load systolic and diastolic blood pressure less than 25 %). The participants were term-born, with normal birth weight, and were not receiving any medication at the time of the examination. The blood and urine tests of subjects in the reference group were within the normal range. Family history of adolescents qualified for this group did not reveal hypertension or other cardiovascular diseases.

Inclusion criteria were as follows: primary arterial hypertension (verified by ABPM—mean systolic or diastolic daytime or nighttime blood pressure levels greater than/or equal to the 95th percentile for age, sex and height, and load systolic or diastolic blood pressure greater than 25 % [13]), normal clinical examination, renal ultrasound findings and electrocardiogram, normal levels of thyroid-stimulating hormone (TSH), and creatinine and urinalysis within normal range.

The following exclusion criteria were used: heart failure, diabetes mellitus, renal or hepatic dysfunction, hematological disease, systemic inflammatory conditions, autoimmune diseases, secondary forms of hypertension, subjects treated with antihypertensive agents and medications known to affect serum LC levels and blood pressure values.

The protocol was approved by the Bioethics Committee of The Medical University of Białystok in accordance with the Declaration of Helsinki. Informed consent was obtained from parents of all participants and children older than 16 years of age.

For all subjects, careful clinical histories were taken and physical examinations were performed. Body weight and height were measured using a balance beam scale and pediatric wall-mounted stadiometer, and body mass index (BMI) was calculated as weight (in kilograms) divided by the square of height (meters squared). BMI *Z* scores, reflecting the SDS for the age and gender appropriate BMI distribution, were calculated using the formula: BMI *Z* scores = (current BMI−50 percentile for age and gender/½(50 percentile−3 percentile for age and gender).

After 12-hours of night fasting, blood samples were taken for the measurement of CRP, basal glucose level, lipid profile, serum creatinine, urea and uric acid levels, and morphology of peripheral blood.

The urine was aseptically collected between 7 and 8 am from morning sample. Urine measurements of Free LC, Total LC and Acylcarnitine were performed in samples frozen at the temperature −70 °C. Patients with pathological leukocyturia were excluded from the examination.

Urinary Free and Total LC were determined by the enzymatic method of Cederblad et al. [14]. Acylcarnitine concentrations were calculated from Total LC minus Free LC concentrations and the Acylcarnitine/Free LC ratio was calculated as: Total minus Free LC/Free LC. Then urine was centrifuged at 2,000×*g* for 10 min (Eppendorf AG Centrifuge 5702R, Hamburg, Germany) and stored at −70 °C before the measurement. The method of Free LC determination is based on the reaction of Free LC with acetyl-CoA, catalyzed by CAT (carnitine acetyltransferase). Free LC with acetyl-CoA forms a CoA-SH, which is determined by reaction with 5,5′-dithiobis-(2-nitrobenzoic acid (DTNB). Increase in absorbance at 412 nm was measured using spectrophotometer Hitachi UV/Vis Model U-2900, Tokyo, Japan. For determination of Total LC, 1 mol/L of KOH solution was added to 150 μL urine and mixed. The mixture was incubated at 56 °C for 1 h (for hydrolysis the carnitine esters), and finally neutralized with 5 mol/L HCl. Liberated Free LC was determined as described above.

Urinary creatinine concentration was used to normalize the Free and Total LC measurements to account for the influence of urinary dilution on its concentration. The urinary levels of creatinine were analyzed by Jaffé’s method. The LC levels were expressed as urinary ratio in micromole per gram creatinine (μmol/g).

Serum creatinine was determined by Jaffe reaction. Morphology of the peripheral blood was assessed by a Coulter analyzer MAXM. Serum cholesterol, HDL-cholesterol, and triglycerides were determined by the enzymatic method and serum uric acid by the colorimetric method using Hitachi 912 (La Roche Company, Japan). Serum glucose was measured with the Integra 800 analyzer.

The estimated glomerular filtration rate (eGFR) was calculated from the Schwartz formula: eGFR = 0.413 × growth (cm)/serum cr. (mg/dL) and was expressed as mL/min/1.73 m^2^.

The 24-h urinary albumin excretion rate (UAER) was analyzed by radioimmunoassay method (RIA). Albuminuria was considered as 24-h UAER >30 mg/24 h. Micro- and macroalbuminuria were defined as 24 h UAER values of 30–300 mg/24 h and >300 mg/24 h, respectively.

Ambulatory blood pressure monitoring (ABPM) was performed using the oscillometric Spacelabs Medical.

The monitors were programmed to measure BP every 15 min during daytime (8:00–22:00) and every 30 min during nighttime (22:00–8:00); however, the periods were corrected according to the subjects’ diaries. Recording started between 8 and 9 a.m. and lasted for 24 h. Recordings with a minimum 80 % of measurement and without breaks longer than 2 h were considered sufficient. The mean SBP and DBP were calculated separately for 24 h and for awake and asleep periods, and also analyzed load systolic (LSBP) and diastolic (LDBP) blood pressure during the day and night. Hypertension (HT) on the basis of ABPM was defined as mean systolic or diastolic daytime or nighttime BP levels that are ≥95th percentile and LSBP or LDBP daytime or nighttime levels more than 25 % [13]. The values were adjusted by gender and body height according to the reference values provided by Wühl et al. [15].

The nocturnal BP decrease (dipping) was calculated as the day-to-night BP difference expressed as percentage of the daytime BP mean. Normal dipping was defined as a fall in the mean systolic or diastolic BP during nighttime of 10 % or more of the corresponding daytime BP.

Each subject or his parent was asked to record the bedtime and time of awakening. After 24 h, the cuff and monitor were removed, and the data downloaded using the manufacturer’s software.

## Statistical methods

Data analysis was performed using a computer program Statistical 10.0 PL. Discrete variables were expressed as counts (percentage), whereas continuous variables as median and range, unless stated otherwise. The comparison between the two groups was done with the Chi-square and Fisher exact tests for categorical variables and *t* test for continuous variables for normally distributed data or Mann–Whitney test for the data distributed not normally. Multiple linear regression analyses were performed including the Free and Total LC/cr. ratio as dependent variable, and with serum levels of total cholesterol, uric acid, and systolic blood pressure considered as independent variables. Correlations between LC and Acylcarnitine and other variables (clinical and laboratory parameters) were evaluated using standard methods, i.e., Pearson’s or Spearman’s test accordingly. The value of *p* < 0.05 was considered statistically significant.

## Results

The demographic and clinical data for each group are summarized in Table 1. Ambulatory blood pressure monitoring and laboratory results were successfully collected from 112 adolescents. Of all studied subjects, 64 were hypertensive (HT) and 48 subjects with white-coat hypertension (reference group). The median age did not differ between groups. Males were more frequently affected with HT than females, consistently with the available reports in the area [16]. In the examined group, 46 subjects (71.9 %) were males (M) and 18 (28.1 %) were females (F), whereas more girls (*n* = 27, 56.3 %) than boys (*n* = 21, 43.7 %) were found in the reference group.

**Table 1:** Anthropometric, clinical and metabolic characteristics of examined group (HT) and reference group (*R*)

	White coat hypertension *N* = 48	Hypertension *N* = 64	*p*
Age (years)	15.3 (10.5–18)	16.60 (12–17.9)	NS
Gender (M/F)	21/27	46/18	<0.01
BMI (kg/m^2^)	20.70 (14.06–32.99)	24.38 (16.65–35.84)	<0.01
BMI *Z* score	0.76 (−1.97 to 7.07)	1.79 (−1.62 to 8.23)	<0.05
RBC (million)	4.91 (3.99–5.66))	5.25 (4.12–5.97)	<0.01
Hb (g/dL)	13.85 (11–16)	15.3 (10.8–17.9)	<0.01
Glucose (mg/dL)	89 (70–102)	91 (78–103)	NS
ALT (U/L)	15 (10–40)	19 (9–52)	<0.05
AST (U/L)	22 (14–41)	21 (14–42)	NS
CRP (mg/dL)	0 (0–0.4)	0 (0–0.5)	NS
Creatinine (mg/dL)	0.62 (0.38–1.13)	0.76 (0.37–1.46)	<0.01
Uric Acid (mg/dL)	4.93 (2.19–7.84)	6.74 (4.28–8.89)	<0.01
Triglycerides (mg/dL)	88 (35–238)	102.5 (42–231)	NS
Cholesterol (mg/dL)	152 (112–212)	165 (116–240)	NS
HDL (mg/dL)	50 (39–76)	50 (33–76)	NS
TG/HDL	1.80 (0.49–5.41)	2.35 (0.76–7.0)	NS
Mikroalbuminuria (mg/L)	3.70 (1.6–40.9)	4.6 (0.3–79)	NS
eGFR(mL/min/1.73 m^2^)	110.86 (89.63–160.04)	93.77 (89.50–175.25)	<0.01
Free l-carnitine (μmol/L)	13.45 (0.2–89.3)	26.65 (1.5–326.6)	<0.01
Total l-carnitine (μmol/L)	17.75 (1.0–88.8)	35.7 (2.6–458.5)	<0.01
Acylcarnitine (μmol/L)	3.83 (0.2–37.2)	6.2 (0.3–131.9)	0.06
Free l-carnitine/cr. (μmol/g)	9.57 (0.34–94.91)	21.63 (0.87–208.96)	<0.05
Total l-carnitine/cr. (μmol/g)	17.18 (1.2–111.88)	30.10 (1.26–293.35)	<0.05
Acylcarnitine/cr. (μmol/g)	4.43 (0.15–47.91)	5.06 (0.16–84.39)	NS

All values are medians. Values given in parenthesis are the range—unless stated otherwise

The body height, weight and BMI of HT children and adolescents were higher as compared to those in the reference (*p* < 0.01). Median BMI *Z* score in the examined group was 1.79 (range −1.62 to 8.23) and was higher in comparison with the references median (*p* < 0.05). Thirty-seven subjects (60.9 %) from the HT group were classified as overweight or obese.

We found statistically significant higher serum creatinine level, red blood cell (RBC) count, and serum hemoglobin level (Hb) (*p* < 0.01) in hypertensive patients when compared to reference group; however, it was probably caused by male predominance in this group.

Similarly serum alanine transaminase (ALT) was significantly higher in HT group (median 19 IU/L; range 9–52 IU/L) when compared to reference group (median 15 IU/L; range 10–40 IU/L) (*p* < 0.05); however, the values were within the reference limits.

We found significant differences in Free LC and Free LC/cr. ratio, Total LC and Total LC/cr. ratio and also in serum level of uric acid and creatinine between HT subjects and healthy reference.

**Fig. 1:**
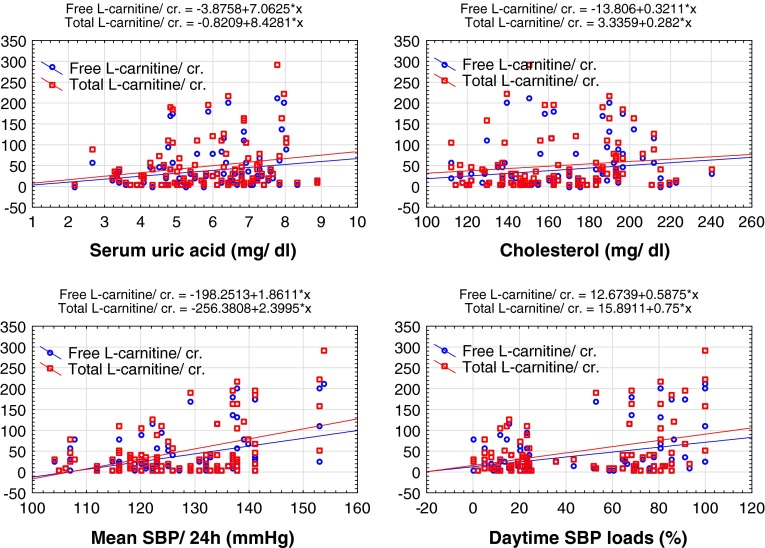
Linear regression analysis demonstrating the relationship between Free l-carnitine/cr., Total l-carnitine/cr. ratio and **a** serum uric acid level, **b** serum cholesterol level and **c** mean systolic blood pressure during 24 h, **d** daytime SBP loads

We performed single regression and correlation analyses of urine parameters with anthropometric, clinical and metabolic measurement and found a significant correlation between the urine Free LC/cr., Total LC/cr. ratio and serum uric acid level (*r* = 0.22, *p* < 0.05; *r* = 0.20, *p* < 0.05, respectively) and serum cholesterol level (*r* = 0.25, *p* < 0.05; *r* = 0.21, *p* < 0.05, respectively) (Fig. 1). The Free LC, Total LC and Acylcarnitine levels were not correlated with age, height, body weight, BMI, hemoglobin concentration, RBC, serum alanine transaminase, aspartate transaminase (AST), serum creatinine levels, serum levels of triglycerides, HDL-cholesterol and LDL-cholesterol.

According to the 24 h ABPM, we found that all the ABPM parameters, except nighttime SBP and DBP load, were significantly higher in children and adolescents with primary hypertension than in the reference group.

We also analyzed the relationship between Free LC, Total LC and Acylcarnitine and the parameters derived from ABPM. We found a positive correlation between Free LC/cr. ratio, Total LC/cr. ratio, Acylcarnitine/cr. ratio and mean SBP/24 h (*r* = 0.27, *p* < 0.05; *r* = 0.30, *p* < 0.01; *r* = 0.24, *p* < 0.05, respectively), Free LC/cr. ratio, Total LC/cr. ratio, Acylcarnitine/cr. ratio and mean SBP during the daytime (*r* = 0.27, *p* < 0.05; *r* = 0.29, *p* < 0.01; *r* = 0.22, *p* < 0.05, respectively), and Free LC/cr. ratio, Total LC/cr. ratio and daytime SBP loads (*r* = 0.23, *p* < 0.05; *r* = 0.24, *p* < 0.05, respectively) (Fig. 1).

The factors that were found to have significant correlation with the urine Free LC/cr. ratio and Total LC/cr. ratio in the single regression analyses were used as explanatory variables to create the multiple regression models. To reduce the impact of multicollinearity, we removed some correlated variables. Next, we eliminated a few more non-significant (in the model) variables and created two models shown in Tables 2 and 3. In the first model, remaining three parameters (serum uric acid, cholesterol and mean systolic blood pressure during daytime—SBP/day) accounted for more than 50.7 % of the variations in Free LC/cr. ratio level (*R* = 0.257, *p* < 0.001) (Table 3). In the second model, also remaining same three parameters accounted for more than 53.7 % of the variations in Total LC/cr. ratio level (*R* = 0.288, *p* < 0.001).

**Table 2:** Multiple linear regression analysis of Free l-carnitine/cr. ratio

	Coefficient	SE	95 % CI	*p* value
Serum uric acid	−10.735	4.843	(−20.388; −1.08)	0.029
Cholesterol	0.344	0.207	(−0.06; 0.736)	0.101
SBP/day	1.942	0.4967	(0.952; 2.93)	0.0002

**Table 3:** Multiple linear regression analysis of Total l-carnitine/cr. ratio

	Coefficient	SE	95 % CI	*p* value
Serum uric acid	−13.834	5.526	(−24.85;−2.81)	0.0146
Cholesterol	0.257	0.236	(−0.21; 0.72)	0.2795
SBP/day	2.513	0.567	(1.38; 3.64)	0.00003

## Discussion

In this cross-sectional study, the urinary excretion of Total and Free LC was significantly higher in hypertensive adolescents in comparison to teenagers, in whom diagnosis of hypertension was not confirmed in 24-ambulatory blood pressure monitoring (reference group—white coat hypertension). Other important findings were positive correlations between Free LC/cr., Total LC/cr. ratio and serum uric acid level, serum cholesterol level and systolic blood pressure. It is interesting to note that we did not find a correlation between LC and serum creatinine levels and eGFR. In this study, serum creatinine levels were higher in children and adolescents with hypertension than in the reference group. Probably, it was due to the significant domination of males in the examined group. After gender adjustment, the difference in serum creatinine levels was not statistically significant. A large and growing body of literature has investigated that primary hypertension in adolescents is more common in males than females [16]. This observation was also confirmed in our report.

In recent years, there has been an increasing amount of literature on the role of LC in hypertension, metabolic syndrome and chronic kidney disease. The mechanisms underlying the effects of LC in cardiovascular diseases are not well clarified. Miguel-Carrasco et al. [17] demonstrated in a rat model that chronic administration of LC reduces blood pressure and attenuates the inflammatory process associated with arterial hypertension. Interesting observations were done by Omori et al. [18] who showed that LC not only had antihypertensive effect but its supplementation also attenuated cardiac fibrosis by increasing prostacyclin production through arachidonic acid pathways.

Studies in humans have demonstrated that LC supplementation may be effective in reducing arterial blood pressure in hypertensive patients [8]. Study by Mate et al. [19] showed that LC had a similar antihypertensive effect to captopril. However, Muniyappa [20] stated that oral LC supplementation could not achieve the plasma carnitine levels required to increase skeletal muscle carnitine concentration.

In reviewing the literature, no data were found on the urine excretion of LC and its derivatives in hypertensive patients. Kidneys play a pivotal role in the establishment and maintenance of carnitine homeostasis. As LC is not systemically metabolized, with the exception of reversible esterification to its acyl derivatives, the primary route of elimination is via renal excretion. LC is a small, polar molecule that does not undergo protein binding, and is freely filtered at the glomerulus such that its filtration clearance is equal to the glomerular filtration rate [21]. After glomerular filtration, it is reabsorbed by 98–99 % in the proximal tubule returned into the bloodstream [9, 22, 23]. Renal carnitine reabsorption is accomplished by OCTN2, a sodium-dependent carnitine carrier expressed in the proximal tubules [24, 25]. The efficiency of the tubular reabsorption of LC increases as dietary intake decreases to maintain circulating LC concentrations within a normal range. On the other hand, when LC concentrations increase, for example with exogenous administration, reabsorption decreases as OCTN2 transporters become partially saturated and greater urinary loss results [26]. It is important to note that LC and its acyl derivatives are both transported via OCTN2. LC is preferentially reabsorbed over its esters such that renal clearance of acylcarnitine is 4–8 times higher than that of LC [26]. Ahmad et al. [21] showed that urine contains 46 % LC, 29 % short-chain acylcarnitines and 16 % long-chain acylcarnitines and it varies considerably from that of plasma. Interestingly, Mancinelli et al. [27] demonstrated that LC and acetyl-LC undergo significant interconversion within the kidney, and the clearance of these generated compounds was approximately ten times higher than LC and acetyl- LC filtered directly from the circulation. It might indicate a possibility of renal secretion of these intracellularly formed compounds.

There are limited data of the urine carnitine excretion in hypertension. Foster et al. [12] reported that in spontaneously hypertensive rats, elevated serum carnitine was associated with increased urinary excretion of carnitine. These findings suggest that the most likely mechanism for increased serum carnitine is significantly increased carnitine synthesis by the liver, with decreased reabsorption by proximal tubule. In our hypertensive patients, serum alanine transaminase was significantly higher, but still within the limit and no correlations with urine LC were found.

In our study, we found positive correlations between Free LC, Total LC, Acylcarnitine and mean SBP/24 h, mean SBP during the daytime and daytime SBP loads. Data from the literature indicate that hypertension leads to the kidneys damage through activation of the renin–angiotensin system (RAS).

Some authors indicate an antioxidant effect of LC in different conditions that are all characterized by an increase in the systemic oxidative stress [28–30]. In recent years, the role of RAS in the antioxidant effect of LC has become a focus of interest to researchers. In experimental study, Zambrano et al. [10] indicated that LC was able to reverse the up-regulation of ACE and AT1 receptor in renal cortex. A reduction in plasma ACE activity has been previously reported in fructose-fed hypertensive rats treated with LC [31]. All these findings suggest an antioxidant effect of LC in renal hypertension mediated by the RAS. This observations might support the use of LC to prevent hypertension. Still little is known about bioavailability of oral LC. Also the relevance of achieved plasma levels in explaining its mechanism of action is questionable.

Another point which should be discussed is positive correlation between Free LC, Total LC and serum uric acid levels. Many large epidemiologic studies have demonstrated that elevated SUA level is associated with HT [1–5]. Increased uric acid level has been reported to induce HT by activating the RAS. Corry et al. [32] reported that uric acid stimulated the major components of the vascular RAS, including the stimulation of both angiotensinogen and angiotensin II production. Also a large and growing body of literature has investigated that hyperuricemia might be a risk factor for renal dysfunction [33]. Mechanism of hyperuricemic nephropathy is still a matter of debate. Previous studies demonstrated uric acid-induced endothelial dysfunction and local inflammation in the kidney as major mechanisms of renal disease [2, 32–35]. However, novel finding of Ryu et al. [36] strongly suggests that uric acid also has direct effects on renal tubules. In reviewing the literature, no data were found on the association between serum uric acid and urine carnitine.

The results of this study do not explain the increased urine levels of LC, because the renal excretion fractions for LC and its derivatives were not determined. However, as LC administration was excluded, the most likely reason for excessive urinary loss was disturbed renal tubular reabsorption. It is possible to hypothesize that in hypertensive teenagers subclinical kidney dysfunction occurs. It is proposed that studies examining the concurrent plasma and urine concentration of LC and correlation with acknowledged proximal tubular markers are needed.

Finally, a number of important limitations need to be considered. Firstly, this study is limited by its relatively small study population and cross-sectional nature. Secondly, the reference group was not a group of healthy teenagers, but a group of patients, in whom hypertension was not confirmed in 24-h blood pressure monitoring, so they are considered white coat hypertension group. Thirdly, there were the evident differences between the studied groups: hypertensive group consisted of adolescent males mainly with high BMI *z* score and reference group included white coat hypertension group of slim females. Fourth, we did not measure the concentration of LC in serum during that time.
